# Supplementary material for: Income inequality, voters’ support for public spending and the size of the welfare state. A simple political model
Source: PLoS One. 2022 Nov 9;17(11):e0277256. doi: 10.1371/journal.pone.0277256 (PMC9645617; doi:10.1371/journal.pone.0277256)
Supplement: S1 Appendix — (PDF) [file pone.0277256.s001.pdf]

Appendix: Conservative majority

A majority of conservatives ($n_{RL}+n_{PL}<1/2$) makes both parties propose their optimal policy $\underline{\alpha}$. In this case, a larger proportion of public resources is used to finance conservative goals such as border enforcement policies. This scenario generates greater casuistry than the previous scenario regarding voters' preferences over the tax rate. As public resources are mostly used to finance their preferred public good, conservative voters might prefer higher taxes than liberal voters. The following lemma characterizes all possible orders of voters' optimal tax rate depending on the level of income inequality and the conservative voters' support for government intervention.

Lemma 2 *There are four orders of voters' optimal tax rate such that:*

Case 1.A. $\tau^*_{PL} > \tau^*_{RL} \geq \tau^*_{PC} > \tau^*_{RC}$ *iff* $\beta \leq \frac{\underline{\alpha}}{1-\underline{\alpha}}\left(\frac{y_P}{y_R}\right)^2$.

Case 1.B. $\tau^*_{PL} > \tau^*_{PC} \geq \tau^*_{RL} > \tau^*_{RC}$ *iff* $\beta \in [\frac{\underline{\alpha}}{1-\underline{\alpha}}\left(\frac{y_P}{y_R}\right)^2, \frac{\underline{\alpha}}{1-\underline{\alpha}}]$.

Case 2.A. $\tau^*_{PC} > \tau^*_{PL} \geq \tau^*_{RC} > \tau^*_{RL}$ *iff* $\beta \in [\frac{\underline{\alpha}}{1-\underline{\alpha}}, \frac{\underline{\alpha}}{1-\underline{\alpha}}\left(\frac{y_R}{y_P}\right)^2]$.

Case 2.B. $\tau^*_{PC} > \tau^*_{RC} \geq \tau^*_{PL} > \tau^*_{RL}$ *iff* $\beta \geq \frac{\underline{\alpha}}{1-\underline{\alpha}}\left(\frac{y_R}{y_P}\right)^2$.

Proof: Consider that there is a majority of conservative voters $n_{RC}+n_{PC} \geq 1/2$. Then both parties propose $\underline{\alpha}$ in the second stage of the political game. From (2.1) and (2.2) it is straightforward that $\tau^*_{PL}(\underline{\alpha}) > \tau^*_{RL}(\underline{\alpha})$ and $\tau^*_{PC}(\underline{\alpha}) > \tau^*_{RC}(\underline{\alpha})$. However, which tax rate is bigger is not so obvious in the comparisons between $\tau^*_{RL}(\underline{\alpha})$ and $\tau^*_{PC}(\underline{\alpha})$; $\tau^*_{iL}(\underline{\alpha})$ and $\tau^*_{iC}(\underline{\alpha})$ $i=R,P$; $\tau^*_{RC}(\underline{\alpha})$ and $\tau^*_{PL}(\underline{\alpha})$. In particular, I find three thresholds for β that define four different orders in voter preferences:

$$\tau^*_{RL}(\underline{\alpha}) \geq \tau^*_{PC}(\underline{\alpha}) \Leftrightarrow \beta \leq \beta_0 \text{ with } \beta_0 = \frac{\underline{\alpha}}{1-\underline{\alpha}}\left(\frac{y_P}{y_R}\right)^2$$

$$\tau^*_{iL}(\underline{\alpha}) \geq \tau^*_{iC}(\underline{\alpha}) \Leftrightarrow \beta \leq \beta_1 \text{ with } \beta_1 = \frac{\underline{\alpha}}{1-\underline{\alpha}}$$

and

$$\tau^*_{PL}(\underline{\alpha}) \geq \tau^*_{RC}(\underline{\alpha}) \Leftrightarrow \beta \leq \beta_2 \text{ with } \beta_2 = \frac{\underline{\alpha}}{1-\underline{\alpha}}\left(\frac{y_R}{y_P}\right)^2$$

According to these thresholds, the possible orders regarding the voters' optimal tax rates are the following:

Case 1.A. $\tau^*_{PL} > \tau^*_{RL} \geq \tau^*_{PC} > \tau^*_{RC}$ iff $\beta \in (0, \beta_0]$.

Case 1.B. $\tau^*_{PL} > \tau^*_{PC} \geq \tau^*_{RL} > \tau^*_{RC}$ iff $\beta \in [\beta_0, \beta_1]$.

Case 2.A. $\tau^*_{PC} > \tau^*_{PL} \geq \tau^*_{RC} > \tau^*_{RL}$ iff $\beta \in [\beta_1, \beta_2]$.

Case 2.B. $\tau^*_{PC} > \tau^*_{RC} \geq \tau^*_{PL} > \tau^*_{RL}$ iff $\beta \in [\beta_2, 1)$.

□

Case 1A and 1B are equivalent to Case 1 and Case 2 of Lemma 1. If conservative voters' support for government intervention is high enough, conservatives prefer a larger tax rate than liberals (Case 2A and Case 2B). Case 2A defines a society in which income generates two opposite blocks of voters, with the poor forming a majority more in favor of higher taxes than the rich.

This is because the conservative support for government intervention is not so high that the rich conservative prefers a larger tax rate than the poor liberal voter. If the latter support is extremely high (Case 2B) then ideology determines two opposite blocks of voters with the conservatives forming a majority.

The following proposition characterizes the political equilibria.

Proposition 3 *If there is a conservative majority, i.e.* $n_{RL}+n_{PL} < 1/2$ *the policy implemented in equilibrium is:* $(\tau^*_{PC}(\underline{\alpha}), \underline{\alpha})$ *in Case 1A and 1B,* $(\tau^*_{PL}(\underline{\alpha}), \underline{\alpha})$ *in Case 2A, and* $(\tau^*_{RC}(\underline{\alpha}), \underline{\alpha})$ *in Case 2B.*

Proof: Consider that there is a majority of conservative voters $n_{RC} + n_{PC} \geq 1/2$. Then both parties propose $\underline{\alpha}$ in the second stage of the political game. According to Lemma 2, I have four cases regarding voters' preferences. Given that voters' preferences are single-peaked and parties are office seekers, both parties propose the optimal policy of the median voter according to the median voter theorem. In case 1.A and 1.B the median voter is the conservative poor voter, since $n_{RC} + n_{PC} \geq 1/2$ and $n_{PL} + n_{PC} \geq 1/2$, respectively. In Case 2, the median voter is the conservative poor voter since $n_{PL} + n_{PC} \geq 1/2$. Hence, the policy implemented in equilibrium is $(\tau^*_{RL}(\overline{\alpha}), \overline{\alpha})$ in Case 1, and $(\tau^*_{PC}(\overline{\alpha}), \overline{\alpha})$ in Case 2.

□

Unlike the scenario of a liberal majority, now the political parties' campaigns always please the conservative poor voter if the level of intensity of the conservative voters' support for public spending is low enough (Case 1A and 1B). This is because if Case 1A conditions hold, there is a majority of conservative voters comprising rich anti-tax voters and poor voters with a more moderate optimal tax policy. Similarly, if Case 1B conditions hold, there is a majority of poor voters with a group of liberal voters who are pro-expenditure and a conservative group with a more moderate view on public expenditure. Following the median voter theorem, parties maximize the probability of winning by proposing the preferred policy of the more moderate voter of the majoritarian block, which is the conservative poor voter in both cases.

Among individuals with the same income, if the optimal tax rate for conservative voters is higher than for liberal voters (Case 2A and 2B), the tax rate implemented in equilibrium depends on the relative intensity of conservatives' support for public spending. If this support is not particularly extreme then the tax rate implemented in equilibrium is the optimal one for the poor liberal voter. This is because the poor liberal voter is the most moderate voter of the majoritarian block of poor voters in Case 2A and both parties maximize their chances of winning by proposing that voter's preferred policy. Otherwise, in Case 2B, where conservatives prefer higher taxes than liberals do independently of their income level, the rich conservative becomes the median voter and both parties propose the rich conservative's favorite policy in equilibrium.
