# Supplementary material for: Income inequality, voters’ support for public spending and the size of the welfare state. A simple political model
Source: PLoS One. 2022 Nov 9;17(11):e0277256. doi: 10.1371/journal.pone.0277256 (PMC9645617; doi:10.1371/journal.pone.0277256)
Supplement: Attachment — Submitted filename: Rebuttal letter.pdf [file pone.0277256.s003.pdf]

Dear Prof. Ponti,

We thank you for the opportunity to revise my paper. I have indeed followed all the suggestions made and we think the paper has clearly improved.

I have enclosed a copy of the revised manuscript along with the response to the reviewer.

We look forward to hearing from you.

Sincerely,

Ángel Solano

Reply to Reviewer #1 (second round)

Dear Reviewer,

We would like to thank you for your very useful comments that have improved the paper considerably. We now detail how we address the minor changes you propose.

1. *To the extent that income inequality is no longer considered as an outcome variable, I think there is no need to name it as "pre-tax" income inequality (there is no such a thing as post-tax income inequality in the current version). Therefore, I suggest revising the text and systematically use "income inequality" (as it stands it might be confusing the use of pre-tax income inequality in some parts while just income inequality in others).*

Done. Thank you for your suggestion.

2. *I suggest defining income inequality (using model notation) the first time it is mentioned in the text. Even though it is quite simple, in my view it would help readership considering it is one of the two key variables in the analysis.*

Done. Thank you for suggesting this.

3. *The intuition of Proposition 1 (last paragraph in page 7) should be revised: the liberal rich becomes the decisive voter if there is either little support for public spending among conservatives (for some given income inequality) or low income inequality (again, for some fixed support for public spending).*

Thank you for your suggestion. I clarified this point using Figure 1.

4. *As it stands, results mentioned in the third paragraph in page 8 could be interpreted as derived from a second order derivative in beta hat ("According to my theory, the type of decisive voter is less reactive to changes in income inequality in consolidated liberal democracies."). I suggest clarifying this point.*

Thank you for suggesting this. I clarified that it is an exercise to see how the decisive voter respond to changes in $\overline{\alpha}$. To do this I introduce the Figure 2.

5. *The impact of income inequality on the liberal rich and conservative poor optimal tax rates should be shown in some more detail considering its relevance in the analysis.*

Thank you for your suggestion. I made explicit the effect of income inequality on the liberal rich and conservative poor optimal tax rates. I also extended the whole subsection and introduced Figure 3 to explain in a clearer way the impact of income inequality on the size of the welfare state.

6. *Is it possible to provide and explicit function for beta*? It would be interesting to provide additional details on this threshold*.

Thank you for suggesting this. The explicit function for β^* depends on the size of the variation of income inequality (a reduction of y_P / y_R). An increase of income inequality can be done through a variation of income levels $y_P^{'}$ and $y_R^{'}$ such that $y_P^{'} / y_R^{'} < y_P / y_R$. This increase could rise or lower the total income level from *Y* to *Y'*. Unfortunately, it is not possible to find an explicit expression of β^* depending on the level of income inequality without normalizing either y_P or y_R, and therefore without assuming and effect on Y. However, it is possible to analyze the effect of the size of an increase in income inequality on β^* without using its explicit function. As I mention in the proof of Proposition 2 as the size of the increase in the income inequality is larger both the optimal tax rate for the liberal rich τ_{RL}^* and $\hat{\beta}(\bar{\alpha}, y_P / y_R) = \frac{\bar{\alpha}}{1-\bar{\alpha}} \left(\frac{y_P}{y_R} \right)^2$ become smaller. Also the slope of the optimal tax rate for the conservative poor τ_{RL}^* with respect to β becomes higher. Therefore, using Figure 4, the higher the size of an increase in income inequality the lower the level of β^* (that is the more likely to have an increase in the tax rate implemented). I introduce this result in the text.

7. *Finally, the paper could really benefit from thorough editing. There are complicated sentences and constructions, as well as grammar issues, that make the paper difficult to follow. Just to give one example, the alternate use of "I" or "we"; p. 6, line 1 (The International Social Survey Programme also obtainS…). is another example of this. Other small errors appear throughout (p. 7 "the the intensity of conservatives'…"; p. 9 "(..) foR the case…" etc.).*

Done. Thank you for suggesting this.
