# Supplementary material for: Income inequality, voters’ support for public spending and the size of the welfare state. A simple political model
Source: PLoS One. 2022 Nov 9;17(11):e0277256. doi: 10.1371/journal.pone.0277256 (PMC9645617; doi:10.1371/journal.pone.0277256)
Supplement: Attachment — Submitted filename: rebuttal letter.doc [file pone.0277256.s002.doc]

Dear Prof. Ponti,

We thank you for the opportunity to revise my paper. I have indeed followed all the suggestions made and we think the paper has clearly improved. In particular, I clarify that the objective of the paper is to better understand how pre-tax income inequality affect the size of the welfare state. To do so, in addition to follow all reviewers’ suggestions, I have changed the title of the paper and include a new subsection.

The main result of the model has an important implication to the future empirical strategies to estimate the effect of income inequality in the size of the welfare state. According to this study, any regression to estimate the size of the welfare state should include, among the usual explanatory variables, an interaction term of two variables such as the pre-tax income inequality and the support of public spending among conservatives voters (relative to liberals).

I have enclosed a copy of the revised manuscript along with the response to both reviewers.

We hope that these changes will make the goal of the paper more clear. Otherwise, I am open to change the structure of the paper if you consider it is essential for the sake of clarity.

We look forward to hearing from you.

Sincerely,

Ángel Solano

**Reply to Reviewer #1**

Dear Reviewer,

Thank you for your thoughtful comments on our manuscript. We believe that now the main goal of the paper is clearer. We have rewritten the title, abstract, introduction and conclusions and included a new subsection to account for your concerns.

Let us now detail how we have addressed your specific comments, keeping the same order as in your report:

*1. The author claims in the introduction and conclusions that income inequality depends on support for public spending among conservatives (and income inequality? See for instance, third paragraph in page 3). This is confusing: in this paper income inequality is an exogenous variable which affects, together with the support for public spending among conservatives, the type of decisive voter.*

*The main result should be clarified and better stated. As it stands now it is the combination of two variables (income inequality and preferences for public spending among conservatives) that determines the decisive voter (either liberal if the former is low and the latter is also low, or conservative in case of high income inequality and/or high support for public spending among conservatives). Whether or not the optimal tax rate among liberals is larger than the one among conservatives depends on the combination of the abovementioned variables.* *Therefore, it is not clear how optimal taxes can be compared in absolute terms which seems to be the conclusion derived from Proposition 1 as mentioned in the Abstract, Introduction and Concluding remarks.*

Thank you for your comment. I agree that it is confusing to use the term income inequality as an exogenous variable and an endogenous variable at the same time. I did that because I was referring to the pre-tax income inequality to the exogenous variable (y_P_/y_R_) and to the post-tax income inequality to the endogenous variable. Implicitly, I assumed that the size of the public good devoted to liberal purposes reduces post-tax income inequality (although it is not modeled as an in-cash social transfer to the poor). Of course this is very confusing and I want to thank you to point this out.

Following your advice I stick to the pre-tax income inequality definition (as an exogenous variable) through the whole paper and analyze its effect (joint with effect of preferences for public spending) on the decisive voter. Also I clarify the main point of the paper as you suggest.

However, I also focus on the effect of the exogenous variables in size of the welfare state (given by the tax rate chosen and the share of tax revenue devoted to provide the liberal public good). This is because this paper tries to contribute to give an answer to the puzzle between the empirical findings and the traditional theories regarding the relationship between pre-tax income inequality and the size of the welfare state. To do so I include a new subsection “The size of the welfare state” with a new Proposition, and a new figure to show the relationship between pre-tax income inequality, voters’ support for public spending and the size of the welfare state. I hope that all this changes helps to make the main points of the paper clearer.

2. *I suggest clarifying what “ideology” means in the abstract while first introducing the term.*

Done. Thank you for your comment.

3. *Also related to this point, I would stick to the “support for public spending” term while referring to ideology and differences between liberal and conservatives: “support for government intervention” might be confusing as government intervention can also be measured through tax size (which is also another endogenous variable in the model)*

Thank for suggesting this. I agree with you and I have done this through all the text.

4*. In my view, the author should add some references to better justify claims related to the strategies followed by rightist parties to attract poor voters (first paragraph in the Introduction)*.

Done. Thank you for your comment.

5. *I suggest moving to the model justifications and empirical support for ideology (why liberals vs conservatives) and the lower support for public spending among conservatives*.

Done. Thank for suggesting this.

6. *The selection of the scenario where there is a liberal majority deserves additional discussion. I suggest better justifying it, by mentioning empirical evidence.*

Done. Thank you for your comment.

7. *In addition, the intuition of results in the other scenario should be commented in the main text to discuss the extent to which the main result in the paper depends on this assumption.*

Done. Thank for suggesting this.

8. *A definition of a threshold β ̂((α,) ̅y_P/y_R)=α ̅/(1-α ̅ ) 〖(y_P/y_R )〗^2 would facilitate readership.* *The discussion of proposition 1 should then be re-written, together with comments on comparative statics around this threshold.*

Done. Thank you for your comment.

9. *Could figures 1 and 2 be done by using the same definition of income inequality used in the rest of the paper?*

Yes I did this with Figure 1. However, and thanks to the definition of the new threshold function *beta hat*, we do need to use Figure 2 to explain how affect the share of tax revenues devoted to liberal policies to the decisive voter. Following the referee’s advice we provide this explanation using the threshold proposed.

Minor comments:

*I suggest being consistent with the use of “&” or “and” in the citing references.*

Done

*Figure 1 is plotted for “some” alpha between 0.25 and 0.75 (i.e., the parameter is fixed).*

Thank you for this comment. Ie add that Figure 1 pictures the decisive voter for the case of *α* upper bar =0.75 which implies that *α* is between (0,25, 0,75) (given that *α* lower bar plus *α* upper bar equals to one)

*There are several typos, for instance: “that” instead of “than” in line 3 page 4; some “.” and “,” should be deleted in proof of Proposition 1.*

Done

**Reply to Reviewer #2**

Dear Reviewer,

Thank you for your thoughtful comments on our manuscript. We believe than now our empirical results are more robust. We have included two new sections (although in an Appendix to not increase too much the length of the manuscript) to account for your concerns.

Let us now detail how we have addressed your specific comments, keeping the same order as in your report:

1. *I find that this paper investigates an extremely important issue for our democratic countries. I think that, however, it does not touch the main issue: why, in the last decades, the system evolved such that the two dimensions (redistribution vs ideology) now interlink more? This is the key question we are facing now.*

I agree with the referee that the question: why, in the last decades, redistribution and ideology interlink more? is very interesting. I also agree that this paper is not addressing this point. However we refer in the text some interesting studies that they do such as Gethin et al. 2021. We use this and other studies to justify our assumptions.

Our paper focus in an old puzzle: according to traditional literature in redistributive politics there is a positive relationship between the size of the welfare state and the level of income inequality. However empirical findings do not confirm this result. We propose and alternative explanation. Moreover, the findings suggest that an interaction term of two variables such as the pre-tax income inequality and the support of public spending among conservative voters (relative to liberal) should be included in any regression to analyze the size of the welfare state.

2 *The model provide an extremely simplified framework in which all the main quantities are exogenously given, and thus no real explanation of the phenomena can be provided. I personally see this peace of research as an interesting starting point for research on this topic but I hardly see it as a full paper.*

I agree with referee that the model is very striped down. However we clarify that there are two endogenous variables which are the tax rate and the share of tax revenues devoted to the liberal public good. Multidimensionality in political competition models makes quite complicate the calculus of equilibria (see Roemer 2001). A solution for this was used in Alesina et al (1999) adopting a two-stage voting process to avoid the problem of multidimensionality when aggregating preferences. I use this method for the sake of clarity.

3. *Considering condition below eq (2.2), if I am not wrong, this should be tau_c(alpha_under)=(beta (1-alpha_under)/alpha^bar) tau_L(alpha^bar). What also happens if beta=1?*

Thank you for this comment. Since α(upper bar) + α(lower bar)=1, we obtain the condition (2.3). Moreover a paragraph is added explaining the results for the case of beta = 1 (see forth paragraph in page 5).

4. *I also think that the author should also improve the understanding of the conditions of the two cases.*

Thank for suggesting this. Using a definition of the new threshold function *beta hat* suggested by the other referee we have rewritten this part. I believe it now less demanding for the reader.

*Reference*

ROEMER, J. E. (2001). *Political Competition: Theory and Applications*. Harvard University Press.
